# High strength tung oil-based adhesive with outstanding wet adhesion for cellulose fiber paper

**DOI:** 10.1039/d6ra06785a

**Published:** 2026-10-07

**Authors:** Chunfeng Yao, Xiaoxue Wu, Feiyang Dong, Yasen Xin, Zongde Wang, Shangxing Chen, Chen Huang, Arthur J. Ragauskas, Xiuxiu Zou

**Affiliations:** a College of Forestry, Jiangxi Agricultural University, Key Laboratory of National Forestry and Grassland Administration on Woody Fragrance and Flavor, Jiangxi Provincial Key Laboratory of Improved Variety Breeding and Efficient Utilization of Native Tree Species Nanchang 330045 China zouxiuxiuxiu@163.com; b College of Life Science, Gannan Normal University China; c Institute of Chemical Industry of Forest Products, Chinese Academy of Forestry Nanjing 210042 China huangchen3127@njfu.edu.cn; d Department of Chemical and Biomolecular Engineering, University of Tennessee Knoxville Knoxville TN 37996 USA; e Center for Renewable Carbon, School of Natural Resources, The University of Tennessee Institute of Agriculture Knoxville TN 37996 USA

## Abstract

The development of bio-based adhesives is hindered due to poor wet adhesion with wet substrate, limited durability, and lack of recyclability. Herein, we report an amphiphilic silane-oil adhesive synthesized *via* a one-step coupling reaction between (3-aminopropyl)triethoxysilane (APTES) and tung oil anhydride (TOA). The amphiphilic molecular architecture of the APTES–TOA adhesive facilitates strong adhesion to cellulose fibers, attributed to the polar interactions of the hydrophilic domains and the water-repellent contribution of the hydrophobic moieties. Thus, the APTES–TOA adhesive endows cellulose fiber paper (CFP) with a tensile strength of up to 117.33 MPa. Mechanistically, interfacial adhesion is governed by ion-diffusion-induced formation of a mobile interphase in wet fiber networks, followed by *in situ* silicification that generates hierarchical mechanical interlocking with cellulose fibers. This dual-stage mechanism significantly enhances the tensile index, tear resistance (2.93 mN m^2^ g^−1^), and folding endurance (12-fold) of CFP. Moreover, the adhesive exhibits excellent reusability, retaining stable bonding performance over at least eight cycles. This work offers a generalizable strategy for designing high-performance, sustainable adhesives for cellulose-based materials and beyond.

## Abstract 2

The development of bio-based adhesives is hindered due to poor wet adhesion with wet substrate, limited durability, and lack of recyclability.

## Graphical abstract

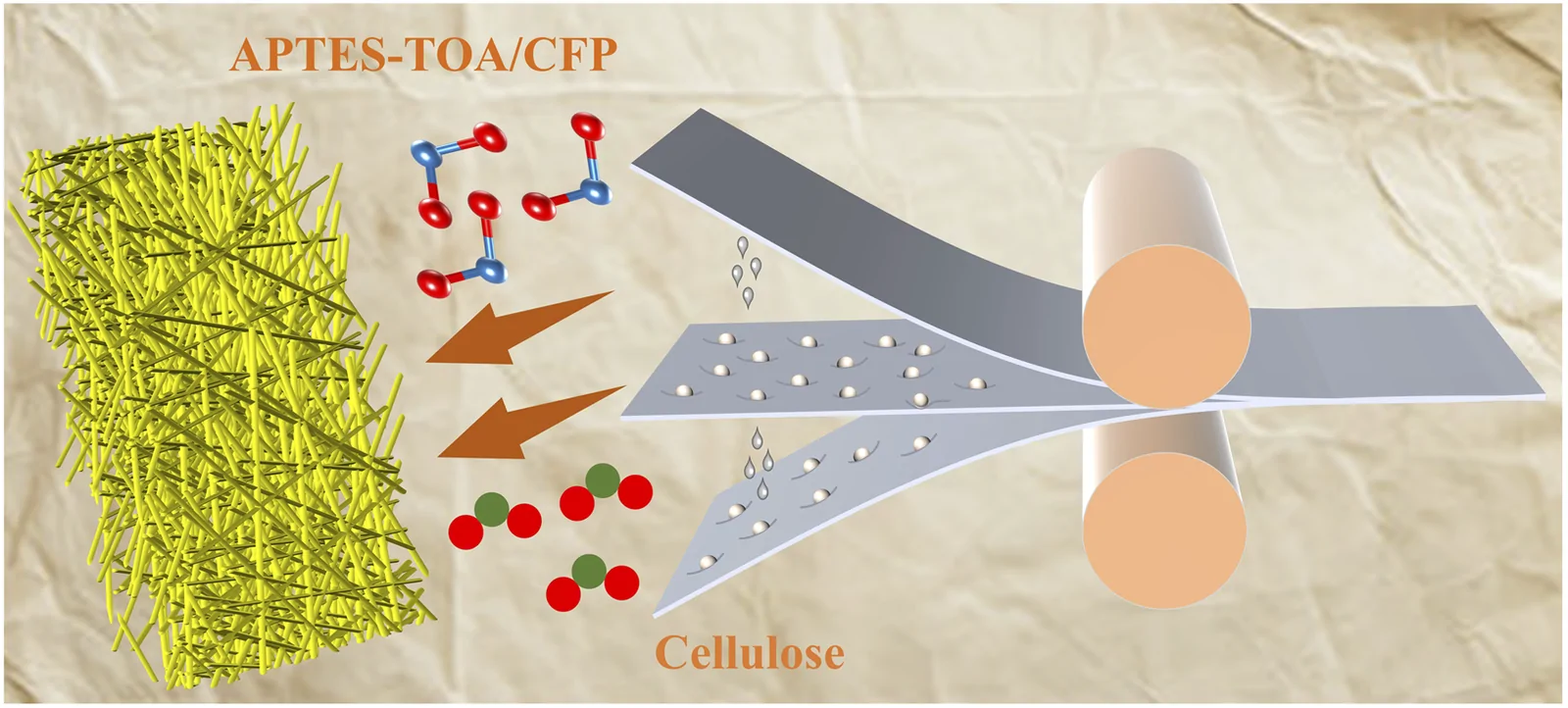

## 1 Introduction

Cellulose, as one of the most abundant natural biopolymers, has been extensively utilized in advanced packaging, construction engineering, printing, clean energy, and emerging sustainable materials.^1–3^ It provides a exercisable platform for developing lightweight materials with desirable toughness, ductility, and cost-effectiveness.^4^ However, the mechanical performance of cellulose-based materials is fundamentally limited by the weak intermolecular interactions (*e.g.* hydrogen bonding and van der Waals forces) between cellulose chains.^5,6^ These non-covalent interactions are easy to be disrupted, particularly under prolonged service conditions, leading to the decrease of strength and durability. Given the critical role of interfacial interactions in determining the mechanical properties of cellulose composites, various adhesives have been used to enhance the binding between cellulose fibers.^7,8^ Currently, synthetic adhesives such as acrylic esters, phenolic resins, epoxies, and alkyl ketene dimers are widely used in the pulp and papermaking industry owing to their low cost, robust adhesion, and excellent water resistance.^9–11^ Huang *et al.*^12^ demonstrated that flocculation effects and interstitial filling played a key role in reinforcing the tensile properties of carbon fiber paper with phenolic resin. Nevertheless, these conventional systems face persistent challenges in balancing mechanical robustness with environmental sustainability. In particular, issues such as the generation of polluted wastewater and process instability caused by adhesive flocculation remain significant concerns.^13^ Therefore, the development of eco-friendly and renewable adhesive systems is highly desirable for next-generation papermaking technologies.

In response, bio-based adhesives derived from renewable resources are attracting increasing attention, such as starch,^14^ proteins,^15^ rosin^16^ and lignin.^17^ Despite notable progress, these materials generally have intrinsic limitations, such as weak adhesion to wet substrates, slow bonding kinetics, and poor processability, which hinder their ability to form conformal and robust interfacial bonding in hydrated fiber networks. Moreover, while strong and rapid adhesion are advantageous for mechanical reinforcement, it often affects the ability to achieve controlled and non-destructive debonding.^18–20^ These challenges underscore the need for innovative design strategies that integrate renewable feedstocks with advanced bonding mechanisms while maintaining scalability and performance. Recently, we reported an adhesive based on tung oil derivative for application in the papermaking industry.^21^ This system relied on electrostatic complexation between tung oil anhydride (TOA) and cellulose within wet fiber networks, followed by the formation of covalent bond during hot pressing, resulting in the enhancement of tensile strength and toughness. Despite these advances, several challenges remain, including the high adhesive dosage required, insufficient cohesion under wet conditions, and the limited applicability of viscous coacervates in flexible systems. These limitations become more obvious in dual-wet interfaces and large-area bonding scenarios. This is mainly due to the intrinsic incompatibility between the hydrophilic cellulose surface and the hydrophobic moieties of TOA.^22^ In addition, the hydrophobic nature of the aforementioned adhesives hinders their removal from substrates, thereby limiting material recyclability.

Herein, we propose a novel composite adhesive design that integrates 3-aminopropyltriethoxysilane (APTES) with TOA to address these challenges. In this system, APTES serves two key functions. On the one hand, it provides hydrophilic compatibility to the hydrophobic TOA. On the other hand, it acts as a mobile phase within the cellulose fiber network. Thus, these functions enhance interfacial interactions and overall cohesion. In whole system, APTES–TOA adhesive effectively infiltrates the porous fiber structure, forming a robust mechanical interlocking network. During hot pressing, APTES undergoes *in situ* self-crosslinking and further reacts with hydroxyl groups on cellulose chains, generating a reinforced crosslinked network (Fig. 1a). Its excellent permeability further expands its applicability to diverse bonding configurations, including both interlayer and intralayer adhesion, thereby significantly increasing the effective bonding area (Fig. 1b). As a result of enhanced interfacial bonding and structural integration, the resulting cellulose fiber paper (CFP) exhibits outstanding mechanical properties—including tensile strength, toughness, tensile index, tear resistance, and folding endurance—which surpass those of previously reported degradable and non-degradable bio-based fiber materials. Notably, this adhesive system also enables the recycling of cellulose substrates through a non-destructive process. This work is expected to provide new insights into the design of sustainable high-performance adhesives and to advance the development of next-generation papermaking technologies and cellulose-based materials.

**Fig. 1:**
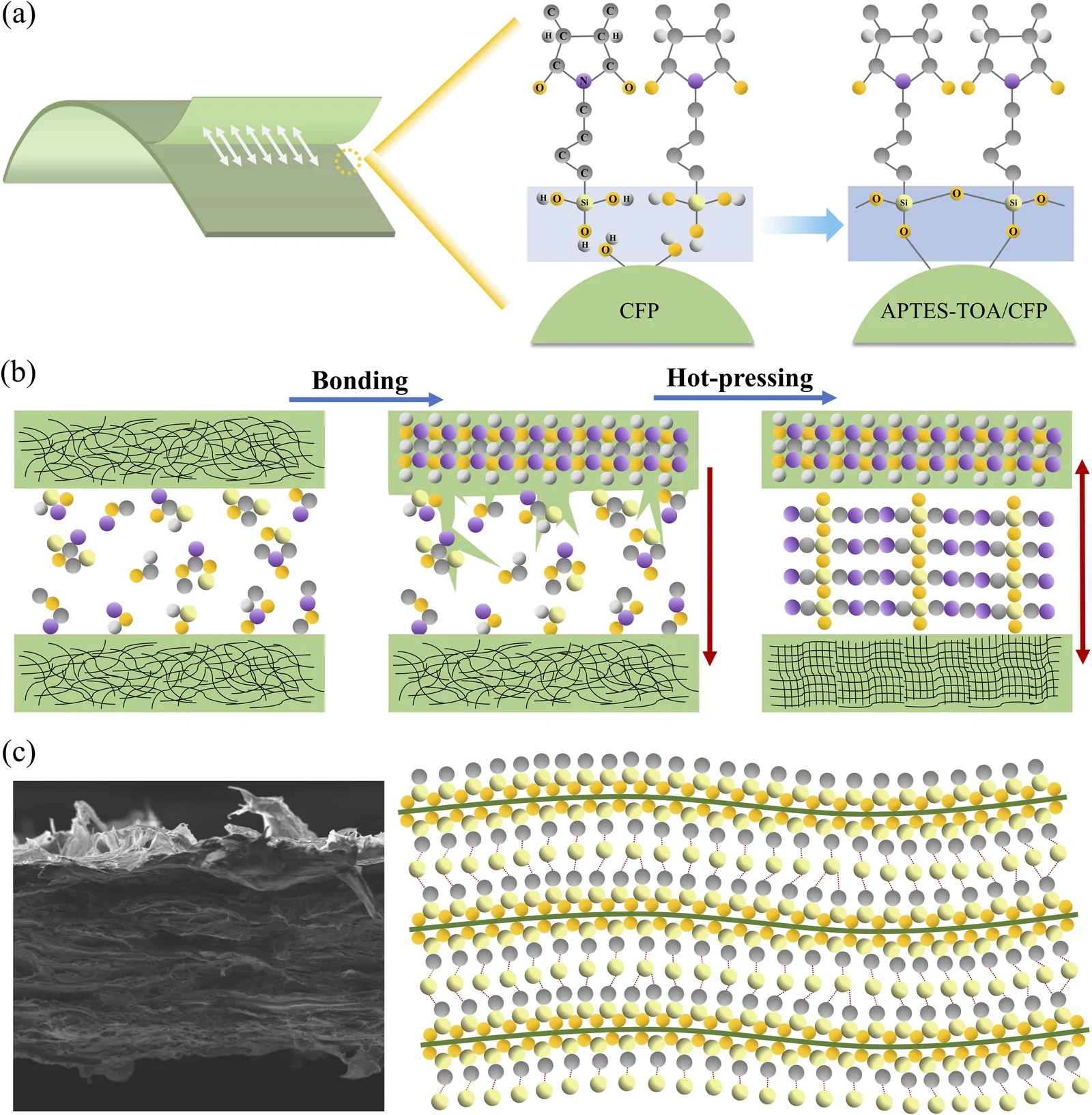
Schematic illustration of the basic concept to achieve the adhesion between APTES–TOA and cellulose (a). Schematic illustration of crosslinking network formation between APTES–TOA adhesive and cellulose fiber through hot-pressing method (b). SEM image of cross-section of APTES–TOA/CFP (c).

## 2 Materials and methods

### 2.1 Chemicals and materials

Cellulose fibers were derived from poplar chemi-thermomechanical pulp (CTMP). Cellulose fibers and TOA (≥99.5%) were supplied by Institute of Chemical Industry of Forest Products, Chinese Academy of Forestry. Acetic acid (≥99.8%, AR), acetone (≥99.5%, AR), sodium chlorite (≥80%, AR), APTES (≥99.5%, AR), benzyl triethyl ammonium chloride (TEBAC, AR) and nonionic emulsifier OP-10 were purchased from Aladdin Chemistry Co. Ltd (Shanghai, China). Cationic starch (DM-933) was purchased from Dongguan Dongmei Food Co., Ltd (Dongguan, China). Nylon mesh with 20 000 mesh was supplied by Foshan Dense-filtration Environmental Protection Technology Co., Ltd (Foshan, China). Cellulose membrane filter with aperture of 0.22 µm and diameter of 50 mm was supplied by Shanghai Xingya purification materials factory (Shanghai, China).

### 2.2 Preparation of APTES–TOA waterborne emulsion

First, 10.00 g of TOA, 6.00 g of APTES, and 0.06 g of TEBAC were added to a 250 mL round-bottom flask. The mixture was stirred at 1500 rpm for 12 h in an oil bath of 108 °C under a nitrogen atmosphere to obtain APTES–TOA. Subsequently, 1.00 g of OP-10 was dissolved in 100 mL of deionized (DI) water under continuous stirring at room temperature for 30 min. Then, 2.00 g of APTES–TOA was slowly added to the above solution. The mixture was further stirred at 1500 rpm in a water bath of 60 °C for 3 h to achieve complete emulsification.

### 2.3 Preparation of cellulose fibers

Cellulose fibers were prepared by selectively removing lignin from poplar CTMP *via* a bleaching process.^23^ Briefly, 2 g of poplar CTMP, 0.75 g of NaClO_2_, 0.5 mL of acetic acid, and 65 mL of DI water were mixed and heated in a water bath at 75 °C for 4 h under continuous stirring. To ensure effective delignification, additional NaClO_2_ (0.75 g) and acetic acid (0.5 mL) were added to the reaction system at 1 h intervals. After the reaction, the mixture was vacuum-filtered and thoroughly washed with DI water and acetone until neutral pH was reached. The resulting fibers were then freeze-dried to obtain cellulose fibers.

### 2.4 Preparation of APTES–TOA/CFP

As shown in Fig. S1, 0.5 g of cationic starch was dispersed in 100 mL of DI water and stirred at 1000 rpm in an oil bath of 90 °C for 30 min to obtain a homogeneous solution. Subsequently, cellulose fibers and the APTES–TOA waterborne emulsion were mixed in a beaker and stirred at room temperature for 5 min. The resulting mixture was vacuum-filtered to obtain wet APTES–TOA/CFP. Pure CFP was prepared using the same procedure without the addition of the APTES–TOA waterborne emulsion. Next, pure CFP was brought to contact with the surface of wet APTES–TOA/CFP to evaluate the adhesion performance on wet surfaces as well as the interfacial adhesion between different layers. The assembled sample was then hot-pressed at 130 °C for 1 h under a pressure of 1.5 MPa using a thermo-compressor to obtain *x* APTES–TOA/CFP, where *x* represents the mass ratio of APTES–TOA in APTES–TOA/CFP.

### 2.5 Tensile property and recycling evaluation

The tensile property of all samples was evaluated using a universal testing machine.^24^ All paper samples were cut into a dumbbell shape with a length of 50 mm and a width of 8.5 mm. Tensile tests were performed by uniaxially stretching the specimens at a strain rate of 20 mm min^−1^. Each sample was tested three times to ensure the reproducibility of the results.

For recycling evaluation, APTES–TOA/CFP was completely disintegrated into individual cellulose fibers in DI water at 90 °C. The obtained fibers were then collected by vacuum filtration and hot-pressed following the same procedure described in Section 2.4. After recycling for 1 to 8 times, the tensile property of the APTES–TOA/CFP samples was measured using a universal testing machine to quantitatively evaluate their recycling performance.

### 2.6 Mechanical properties of cellulose fiber paper

The mechanical properties of CFP and APTES–TOA/CFP, including tensile index, tear index, and folding endurance, were evaluated. According to Chinese Standard GB/T 12914-2008, paper samples were cut into rectangular strips with dimensions of 120 mm × 15 mm, and the tensile index was measured using a tensile strength tester (CE062, L&W, Sweden).^25^ According to Chinese Standard GB/T 455-2002, samples with dimensions of 63 mm × 50 mm were prepared to determine the tear index using a tear strength tester (CE009, L&W, Sweden).^26^ According to Chinese Standard GB/T 457-2008, samples were cut into dimensions of 60 mm × 15 mm, and the folding endurance was measured using a double-chuck folding endurance tester (Frank-PTI 13505, Frank-PTI, Germany).^27^ All measurements were performed in triplicate, and the results were averaged.

### 2.7 Characterization

The interactions between APTES and TOA were determined by proton nuclear magnetic resonance spectroscopy on a spectrometer (^1^H NMR, Avance 400, Bruker, Germany). The structure of APTES–TOA and APTES–TOA/CFP was characterized using X-ray photoelectron spectrometer (XPS, K-Alpha, Thermo Scientific, USA), with a spot size of 400 µm, an energy step size of 1.2 KV. The Fourier transform infrared (FTIR) spectra of all sample were obtained using a FTIR spectrometer (Nicolet iS20, Thermo Scientific, USA), with scanning wavenumbers ranging from 500 to 4000 cm^−1^. The crystal structure of CFP and APTES–TOA/CFP was characterized by using an X-ray diffractometer with Cu Kα radiation (XRD, D8 Advance, Bruker, Germany). The scanning speed was set at 10° min^−1^, and the scanning range was from 5° to 80°. The surface morphology of CFP and APTES–TOA/CFP was examined using a scanning electron microscopy (SEM, SU8220, Japan) at an accelerating voltage of 10 kV, which all samples were coated with gold palladium in a sputter coater. In addition, the elemental analysis was also obtained by a SEM combined with an energy-dispersive X-ray elemental mapping system. Differential scanning calorimetry analysis (DSC, STA 449 F3, Netzsch, Germany) was used to evaluate the thermostability of CFP and APTES–TOA/CFP. All samples were measured under a nitrogen atmosphere scanning from 30 °C to 800 °C at a heating rate of 10 °C min^−1^.

## 3 Results and discussion

### 3.1 Chemical structural analysis

The interactions between APTES and TOA were investigated using ^1^H NMR spectroscopy. As shown in Fig. 2a and b, the ^1^H NMR spectra of APTES and TOA exhibited distinct resonances. In the spectrum of APTES, three prominent peaks at 1.03 ppm, 1.08 ppm and 3.63 ppm were assigned to the CH_3_, NH_2_ and CH_2_ groups, respectively.^28^ In the spectrum of TOA, the signals in the range of 5 to 6 ppm were attributed to the protons of the carbon–carbon double bonds, while additional peaks at 2.31 and 2.76 ppm corresponded to the CH_2_ and CH groups. Compared with APTES, the ^1^H NMR spectrum of APTES–TOA showed the disappearance of the NH_2_ proton signals, indicating that the amino groups of APTES reacted with the anhydride groups of TOA. The chemical structure of APTES–TOA and APTES–TOA/CFP was further characterized by FTIR spectroscopy (Fig. 2d). In the FTIR spectrum of APTES–TOA, the characteristic absorption bands at 1735 cm^−1^ (C=O stretching of TOA) and 1078 cm^−1^ (Si–O–C vibration) confirmed the successful incorporation of both components. Meanwhile, the characteristic peaks of the anhydride groups at 1845 and 1776 cm^−1^ from TOA and the –NH_2_ bending vibration at 1602 cm^−1^ from APTES largely disappeared after reaction. Instead, new absorption bands appeared at 1652 and 1231 cm^−1^, which were assigned to the C=O and C–N stretching vibrations of amide bonds. This result confirmed the formation of amide linkages between APTES and TOA. For APTES–TOA/CFP, characteristic peaks at 1164 and 1078 cm^−1^ corresponding to C–O and Si–O–C vibrations were observed, indicating the successful introduction of APTES–TOA into the cellulose framework. Notably, a new peak at 1022 cm^−1^ attributed to the Si–O–Si stretching vibration suggested the self-condensation of APTES.^16^ The FTIR spectrum of APTES–TOA/CFP was largely similar to that of CFP, indicating that the cellulose backbone remained intact during the modification process. However, the OH stretching peak shifted from 3285 to 3318 cm^−1^ after the incorporation of APTES–TOA, suggesting the formation of hydrogen bonding interactions between cellulose and APTES–TOA.

**Fig. 2:**
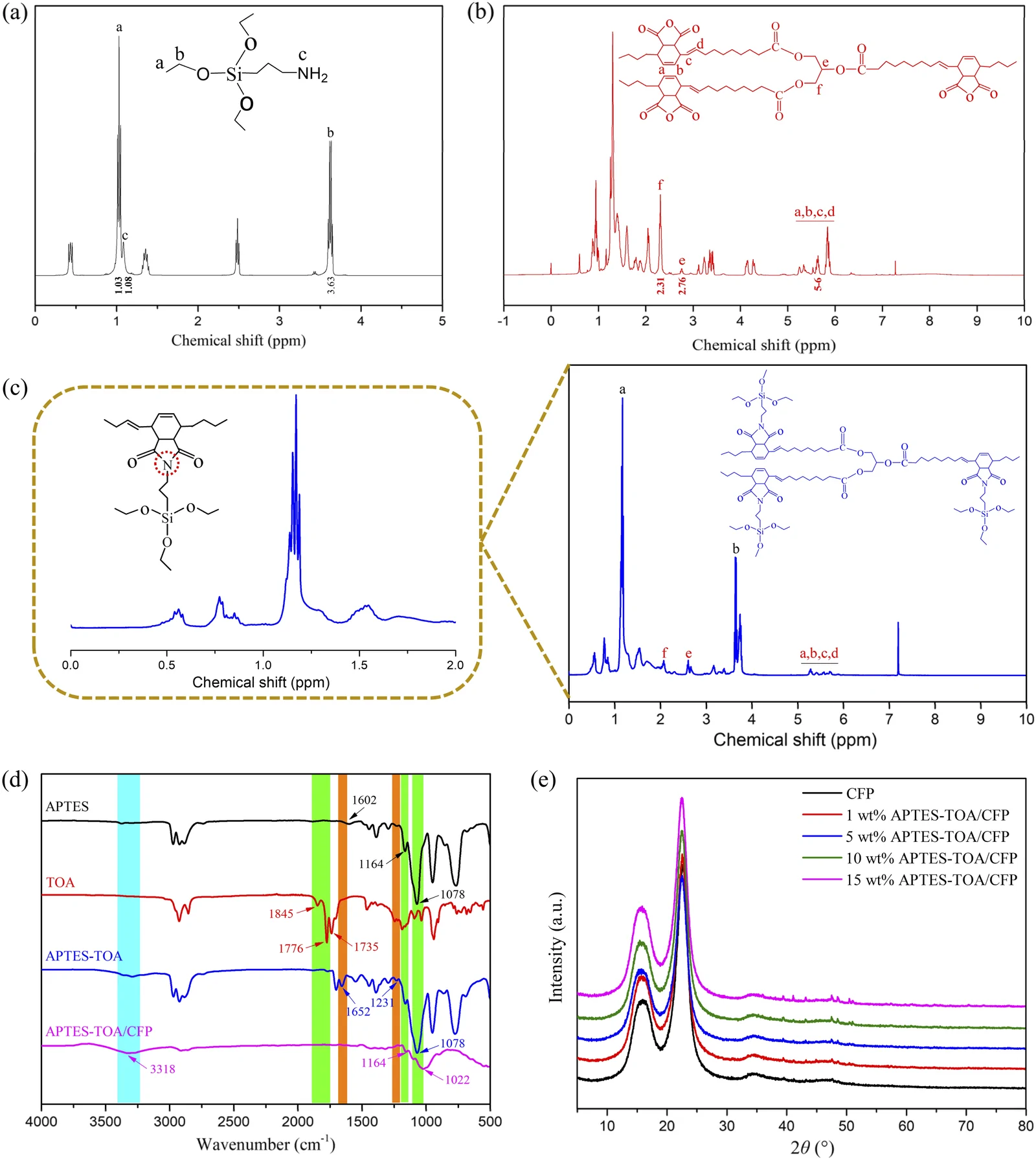
^1^H NMR spectra of APTES (a), TOA (b) and APTES–TOA (c). FTIR spectra of APTES, TOA, APTES–TOA and APTES–TOA/CFP (d). XRD spectra of APTES, TOA, APTES–TOA and APTES–TOA/CFP (e).

To further evaluate the effects of APTES–TOA on the crystalline structure of cellulose, XRD analysis was conducted. As shown in Fig. 2e, CFP exhibited a typical cellulose I structure with diffraction peaks at 15.2°, 15.8°, 22.5°, and 34.5°, corresponding to the (1̄1̄0), (110), (200), and (004) planes, respectively.^29^ After incorporation of APTES–TOA, these characteristic peaks remained unchanged. The crystallinity showed negligible variation, indicating that the ordered crystalline structure of cellulose was well preserved. As shown in Fig. S2, the particle size distribution of the APTES–TOA emulsion (80%) ranged from 400 to 520 nm. Moreover, the average particle size exhibited minimal variation over time, demonstrating the good stability of the waterborne emulsion.

XPS analyses of APTES, APTES–TOA and APTES–TOA/CFP were performed to investigate their surface chemical states. In the O 1s spectrum of APTES–TOA (Fig. 3a), two peaks located at 531.4 eV and 532.8 eV were assigned to C–O and Si–O bonds, respectively. This result was consistent with those observed in the spectrum of APTES. Notably, a new peak at 532 eV corresponding to the N–C=O bond was observed in the O 1s spectrum of APTES–TOA, providing evidence for the formation of amide linkages between the amino groups of APTES and the anhydride groups of TOA.^30^ The O 1s spectrum of APTES–TOA/CFP exhibited a significantly enhanced Si–O signal intensity compared with that of APTES–TOA. This enhancement is consistent with the formation of Si–O–C bonds between APTES and cellulose, although a contribution from APTES self-condensation cannot be excluded. To further clarify the bonding states, the N 1s and Si 2p spectra were examined. In the N 1s spectrum of APTES (Fig. 3b), two distinct peaks at 398.5 and 400.4 eV were assigned to N–H and C–N bonds, respectively. In contrast, the N–H signal disappeared in the spectrum of APTES–TOA, confirming that the amino groups of APTES were consumed during the reaction with TOA. Furthermore, in the Si 2p spectra (Fig. 3c), peaks at 102.1 and 102.8 eV were attributed to C–Si and C–O–Si bonds, respectively. Compared with APTES–TOA, APTES–TOA/CFP exhibited an additional peak at 101.9 eV, which was assigned to Si–O–Si bonds.^16^ These results demonstrate that APTES not only reacted with the hydroxyl groups of cellulose to form Si–O–C linkages, but also underwent self-condensation to generate a highly crosslinked Si–O–Si network.

**Fig. 3:**
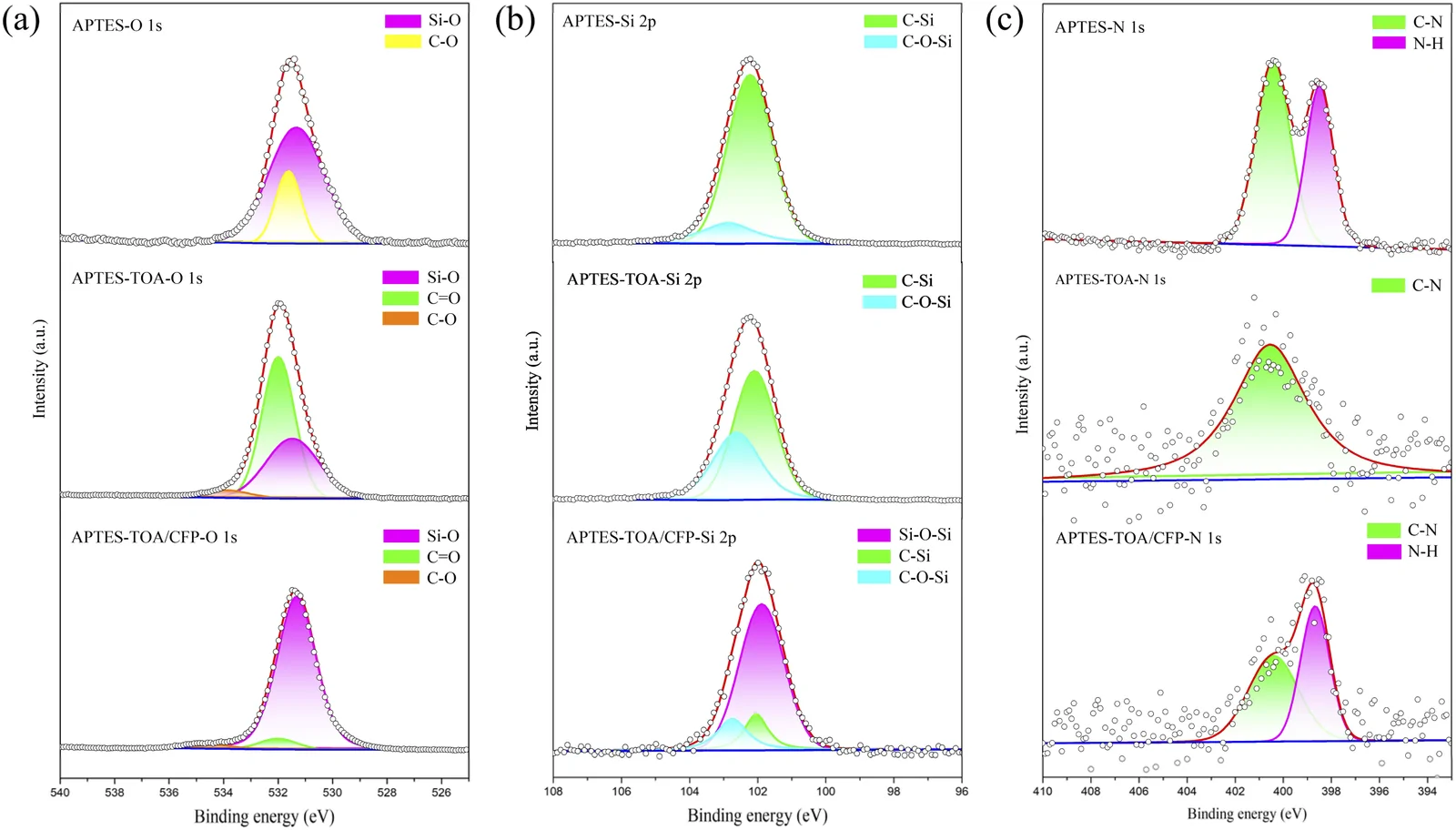
High-resolution O 1s (a), Si 2p (b) and N 1s (c) XPS spectra of APTES, APTES–TOA and APTES–TOA/CFP.

### 3.2 Morphological analysis

The morphology of CFP and APTES–TOA/CFP was examined by SEM to evaluate the loading and dispersion of APTES–TOA on the fiber surface. As shown in Fig. 4a, pure cellulose fibers exhibited a typical fibrous morphology. In addition, the CFP displayed an interpenetrating network formed by entangled cellulose fibers at the microscale. After hot-pressing, APTES–TOA appeared as lamellar nanosheets, while the morphology of the cellulose fibers remained largely unchanged. Notably, the fiber surface was uniformly coated with abundant APTES–TOA nanosheets, which also filled the porous framework of CFP. This hierarchical micro/nanostructure is expected to stabilize the crosslinked network within the CFP system by increasing the contact area and promoting mechanical interlocking.^31^ To further evaluate the dispersion uniformity of APTES–TOA within CFP, elemental mapping of 10 wt% APTES–TOA/CFP was characterized using SEM-EDS. As shown in Fig. 4f–i, C, O, N, and Si elements were uniformly distributed across the surface of APTES–TOA/CFP, which confirmed the good dispersion of APTES–TOA within the CFP.

**Fig. 4:**
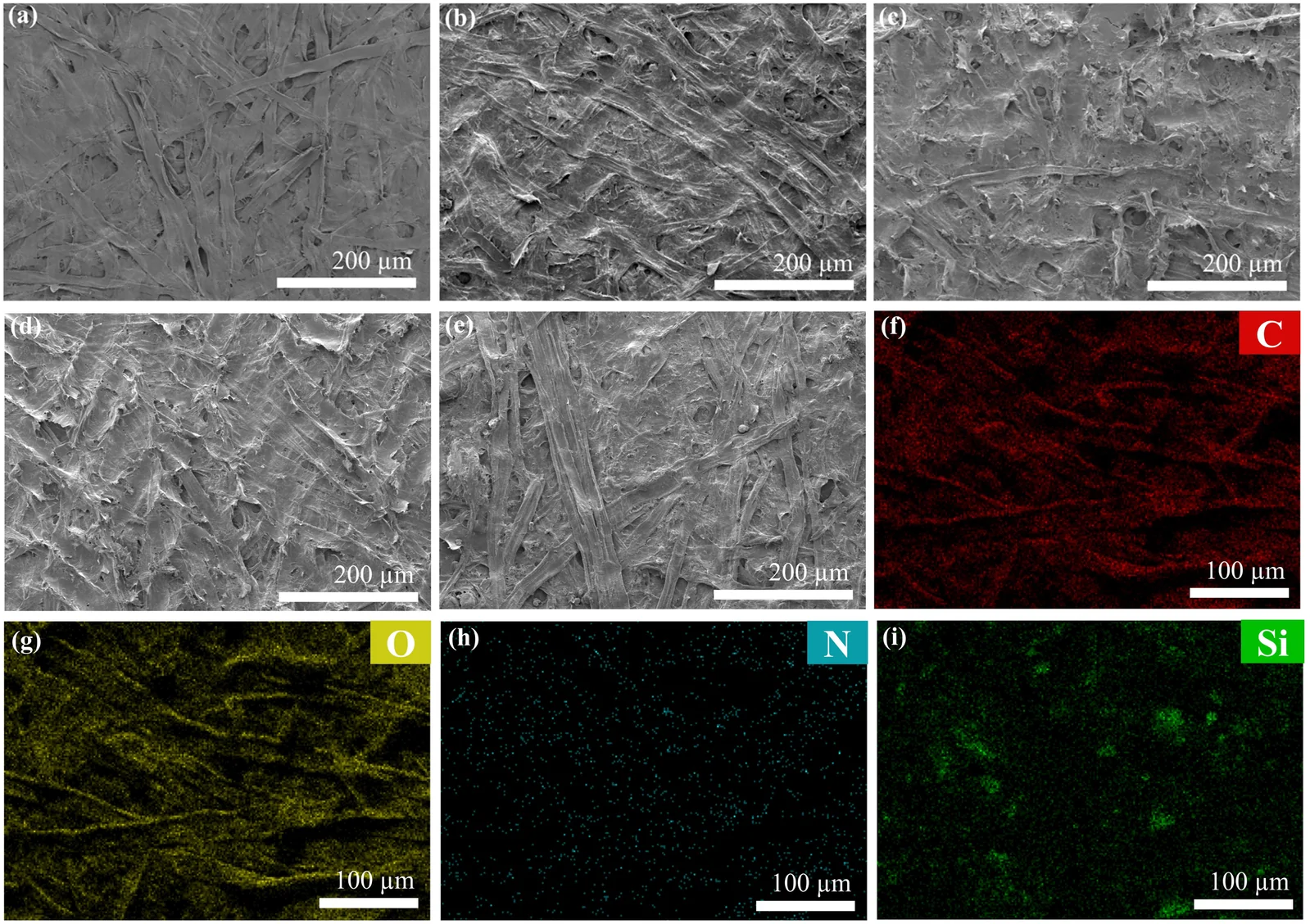
SEM images of CFP (a), 1 wt% APTES–TOA/CFP (b), 5 wt% APTES–TOA/CFP (c), 10 wt% APTES–TOA/CFP (d) and 15 wt% APTES–TOA/CFP (e). Elemental mapping images of 10 wt% APTES–TOA/CFP correspond to C, O, N, Si elements (f to i).

### 3.3 Morphological analysis of cross-section

To investigate the interfacial adhesion mechanism of APTES–TOA adhesive within CFP, the cross-sectional morphology of APTES–TOA/CFP was examined by SEM. As shown in Fig. 5a and b, pure CFP exhibited a loose internal structure with noticeable gaps between cellulose fibers, indicating insufficient inter-fiber bonding and the absence of a continuous network.^32^ To clarify the role of the wet hot-pressing process, a control sample was prepared *via* dry hot pressing. In this case, a clear interfacial separation between the APTES–TOA-containing layer and the pure CFP layer was observed (Fig. 5d and e), suggesting poor interfacial integration. This observation was corroborated by EDS face-mapping analysis (Fig. 5f), which revealed limited penetration of APTES–TOA into the CFP matrix under dry conditions. In contrast, when the wet hot-pressing process was applied, no obvious interfacial separation was observed in the SEM images (Fig. 5g and h). The result indicates that the APTES–TOA adhesive effectively penetrated the CFP network. The presence and distribution of APTES–TOA within the CFP were further confirmed by EDS mapping (Fig. 5i). In the cross-sectional line-scan profiles, N and Si elements were uniformly distributed, demonstrating the effective permeability of the APTES–TOA adhesive. The enhanced penetration behavior can be attributed to two factors. First, water provides an aqueous medium that facilitates the transport and infiltration of APTES–TOA into the porous cellulose network. In the absence of sufficient fluidity, the adhesive remains primarily at the interface, that is evidenced by the weak N and Si signals in the EDS mapping (Fig. 5f). Second, the combination of temperature and applied pressure during wet hot pressing promotes deeper infiltration and interfacial integration, that is reflected by the strong and uniform elemental signals (Fig. 5i). These results indicate that APTES–TOA was effectively integrated into the cellulose fiber network, forming a dense and continuous interconnected structure. Based on these results, a schematic adhesion mechanism was proposed. Under wet hot-pressing condition, APTES–TOA penetrated into the CFP matrix under pressure and adhered to the fiber surface, forming a continuous bonding network (Fig. 5j). The hydrophilic groups of APTES further facilitate infiltration into the fiber network, promoting the formation of a compact and highly integrated interfacial structure (Fig. 5k).

**Fig. 5:**
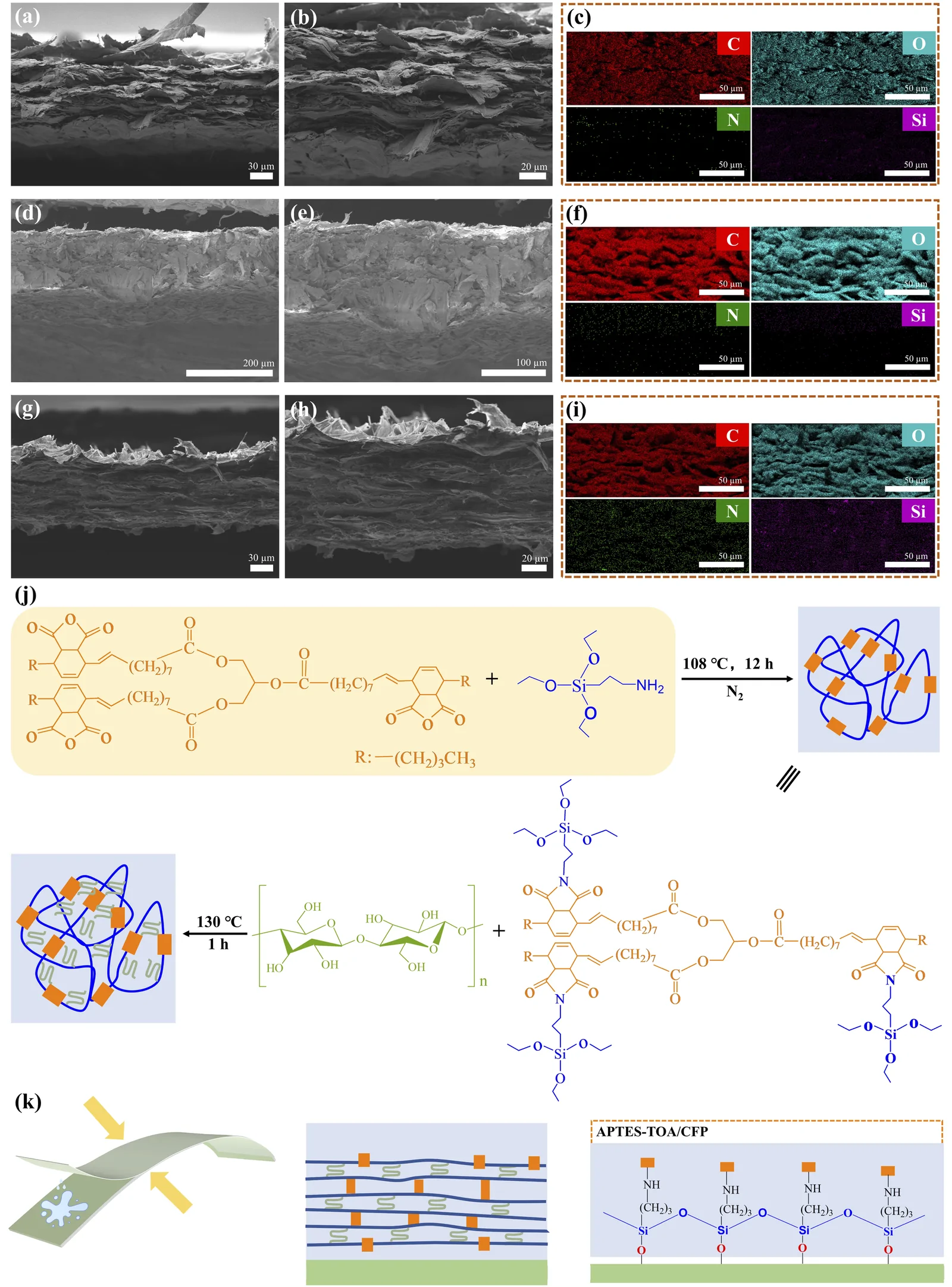
SEM images and elemental mapping images of CFP (a–c), 10 wt% APTES–TOA/CFP of dry state under a hot-pressing (d–f) and 10 wt% APTES–TOA/CFP of wet state under a hot-pressing (g–i). The chemical reaction process of APTES–TOA and CFP (j). Schematic illustration of the interfacial adhesion between APTES–TOA and CFP (k).

### 3.4 Mechanical properties

Excellent mechanical properties are conducive to the broad application of CFP in various fields. The tensile properties of CFP and APTES–TOA/CFP were measured using a universal testing machine. As shown in Fig. 6a, CFP had a tensile strength of 24.48 MPa. In comparison, APTES–TOA/CFP exhibited significantly enhanced tensile performance, and the maximum tensile strength increased from 48.11 to 117.33 MPa with increasing the addition of APTES–TOA. This improvement indicates that the APTES–TOA adhesive significantly enhanced inter-fiber bonding. Consistent with previous studies, the strong interfacial interactions can be attributed to multiple forces, including electrostatic interactions, hydrogen bonding, and covalent bonding.^33^ Furthermore, the effects of APTES–TOA content to the mechanical properties of CFP including tensile strength, elongation at break, and Young's modulus, were investigated. As shown in Fig. 6c, the mechanical strength of APTES–TOA/CFP initially increased and then slightly decreased as the APTES–TOA content increased from 1 to 15 wt%. The optimal performance was achieved at 10 wt%, with a tensile strength of approximately 117.33 MPa, an elongation at break of 6.8%, and a Young's modulus of 3.74 GPa. Notably, the tensile strength increased by nearly fivefold compared with CFP, demonstrating that the incorporation of APTES–TOA significantly enhanced inter-fiber bonding. Compared with previously reported non-degradable and degradable plastic papers, the 10 wt% APTES–TOA/CFP developed in this study exhibits higher tensile strength (Fig. 6b).^34–45^

**Fig. 6:**
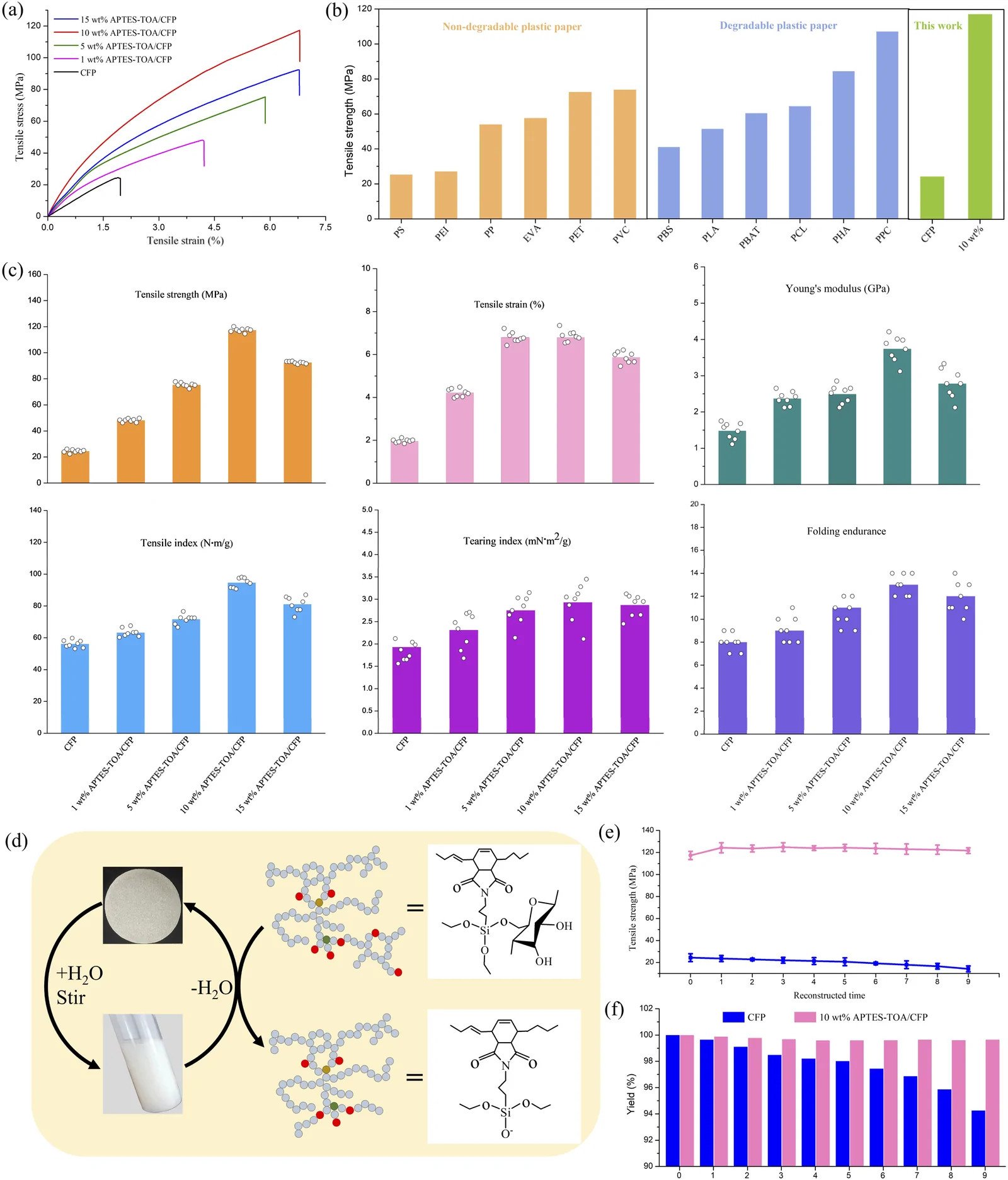
The tensile stress–strain curves of CFP and APTES–TOA/CFP (a). Comparison of the tensile strength of APTES–TOA/CFP with those of nondegradable plastic papers and degradable plastic papers (b). The mechanical properties of CFP and APTES–TOA/CFP including tensile strength, tensile strain, Young's modulus, tensile index, tear index and folding endurance (c). Schematic diagram of recycling of APTES–TOA/CFP (d). The tensile strength (e) and yield (f) of CFP and APTES–TOA/CFP during the cycle period.

The tensile index, tear index, and folding endurance of CFP and APTES–TOA/CFP were further evaluated to assess the overall mechanical performance of the paper materials. As shown in Fig. 6c, the incorporation of APTES–TOA significantly improved three mechanical parameters. Specifically, APTES–TOA/CFP exhibited a maximum tensile index of 94.6 N m g^−1^, a tear index of 2.93 mN m^2^ g^−1^, and a folding endurance of 13-fold, which was markedly higher than CFP (56.12 N m g^−1^, 1.93 mN m^2^ g^−1^, and 8-fold, respectively). The influence of APTES–TOA content on mechanical properties was further investigated. As the APTES–TOA content increased from 1 to 10 wt%, the tensile index, tear index, and folding endurance increased from 63.22 to 94.6 N m g^−1^, 2.31 to 2.93 mN m^2^ g^−1^, and 8 to 12-fold, respectively. This improvement can be attributed to the increase of crosslinking density among cellulose fibers, which strengthens inter-fiber cohesion and adhesion. Compared with previously reported adhesive of fiber paper and commercial adhesives, the bio-based adhesive developed in this study exhibits superior bonding property (Fig. S3).^16,21,46–54^ However, when the APTES–TOA content further increased to 15 wt%, all mechanical properties decreased. This decline was likely due to inhomogeneous solidification and localized aggregation of the adhesive at high contents, which disrupted the uniformity of the crosslinked network and reduce the overall inter-fiber bonding strength.

In addition to strong interfacial bonding, an ideal adhesive should also enable on-demand and non-destructive detachment from adhered substrates. To evaluate the detachment capability and reusability of the APTES–TOA adhesive, the reversible cycling performance of the APTES–TOA/CFP system was investigated (Fig. 6d). The maximum tensile strength of APTES–TOA/CFP increased slightly from 117.33 to 121.69 MPa after eight detachment/recombination cycles, whereas that of pristine CFP decreased markedly from 24.48 to 14.26 MPa (Fig. 6e). This result demonstrated the enhanced durability and reversibility of the APTES–TOA adhesive system. The slight increase in tensile strength was likely due to improved dispersion of the APTES–TOA adhesive within the CFP matrix during repeated cycling. In addition, a negative correlation between fiber yield and the number of detachment/recombination cycles was observed (Fig. 6f). Compared with APTES–TOA/CFP, the fiber yield of CFP decreased more markedly after eight cycles, which was mainly attributed to the loss of fiber fines during the repeated processing.^55^

### 3.5 Thermal stability

TGA was conducted to evaluate the thermal stability of APTES–TOA/CFP. The TGA and DTG curves of CFP and APTES–TOA/CFP are presented in Fig. 7, and the corresponding parameters are summarized in Table 1. Both CFP and APTES–TOA/CFP exhibited three typical stages of thermal degradation.^56^ The first stage occurred below 250 °C, which is mainly attributed to the evaporation of adsorbed water. The second stage corresponded to the primary decomposition process, that shows the highest weight loss rate. For CFP, the major weight loss between 250 °C and 350 °C was attributed to the thermal decomposition of cellulose, with a maximum decomposition temperature (*T*_max_) of 331.88 °C. In comparison, the weight loss of APTES–TOA/CFP in this stage arises from the decomposition of both cellulose and the APTES–TOA adhesive. Notably, *T*_max_ shifted from 331.88 °C of CFP to 358.68 °C of 10 wt% APTES–TOA/CFP, indicating that the incorporation of APTES–TOA significantly enhanced the thermal stability of CFP. The third stage occurred above approximately 400 °C, that is associated with the carbonization of cellulose and APTES–TOA components. In addition, the residual char yield increased from 11.34% for CFP to 22.86% for 10 wt% APTES–TOA/CFP, suggesting enhanced thermal shielding due to the formation of a protective APTES–TOA-derived layer. In contrast, lower APTES–TOA loadings (5 wt%) exhibited a negligible effect on the char yield. Overall, the thermal stability of CFP is significantly improved after the incorporation of APTES–TOA adhesive, which is beneficial for maintaining long-term adhesion performance under harsh environmental conditions.

**Fig. 7:**
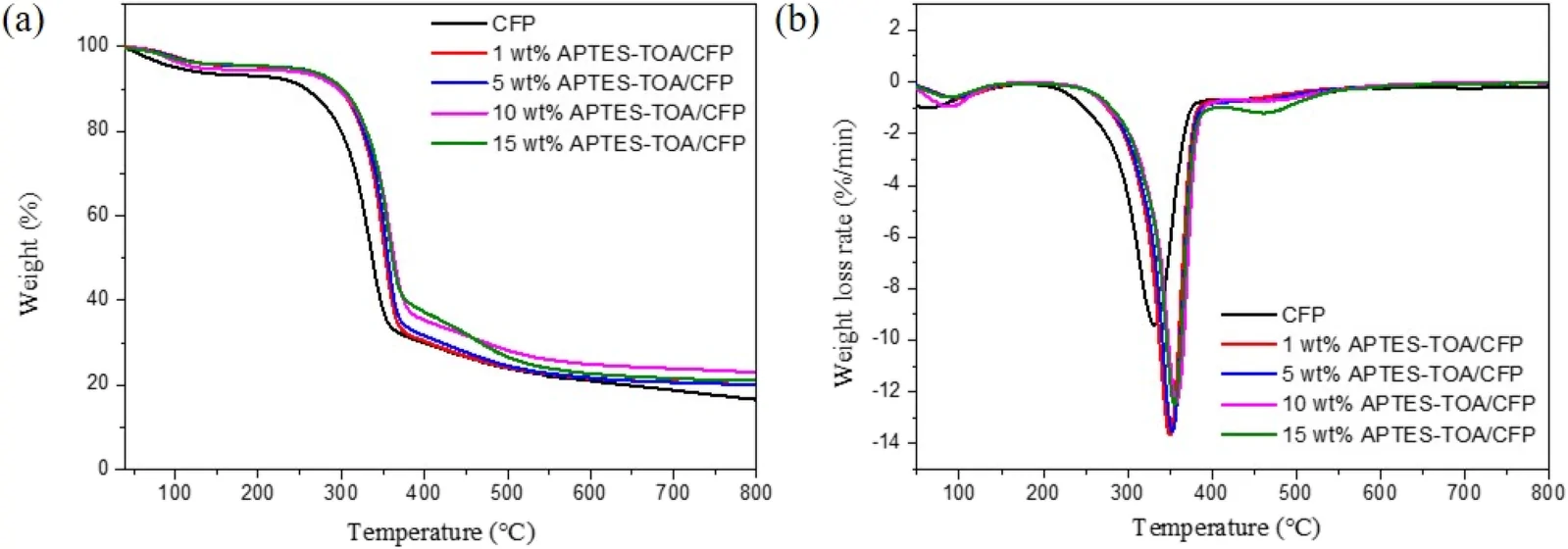
TG (a) and DTG (b) curves of CFP and APTES–TOA/CFP under N_2_.

**Table 1:** Pyrolytic dates of CF paper and TOA/CF paper under N_2_

Sample	*T*_50%_/°C	*T*_max_/°C	Char yield/%
CFP	336.23	331.88	11.34
1 wt% APTES–TOA/CFP	353.01	350.01	20.096
5 wt% APTES–TOA/CFP	356.10	353.92	20.101
10 wt% APTES–TOA/CFP	363.97	358.68	22.86
15 wt% APTES–TOA/CFP	361.31	355.20	20.996

## 4 Conclusion

This study demonstrated the synthesis of a bio-based adhesive by integrating the APTES and TOA. The system featured a modular design in which the matrix and adhesive layer could be independently tailored, offering high flexibility for designing adhesive patches for diverse applications. Importantly, this strategy eliminated the labor-intensive modifications and complex sizing processes typically required for conventional adhesives. To demonstrate its versatility, cellulose fiber networks incorporating varying amounts of APTES–TOA adhesive were fabricated. The incorporation of APTES–TOA into the fiber matrix induced the formation of a dense structure, coupling with a dual crosslinked network consisting of covalent bonds and interfacial hydrogen bonding. This structural design significantly enhanced interfacial interactions between cellulose fibers. During hot pressing, the APTES–TOA adhesive penetrated into the interlayer regions of the fiber network, leading to the formation of APTES–TOA/CFP. The resulting material exhibited excellent mechanical performance, with a tensile strength of up to 117.33 MPa, a tensile index of 94.6 N m g^−1^, a tear index of 2.93 mN m^2^ g^−1^, and a folding endurance of 13-fold, outperforming previously reported non-degradable and degradable plastic papers. Furthermore, the strong bonding network between APTES–TOA and cellulose fibers enabled the adhesive to maintain its tensile strength unchanged after at least eight cycles of use. As a novel bio-based adhesive, APTES–TOA effectively overcomes the limitations of poor reusability and restricted processing conditions associated with traditional adhesives. Overall, this study provides a general and straightforward strategy for achieving strong interlayer and intralayer adhesion in fiber-based materials, thereby broadening the potential applications of bio-based adhesives.

## Author contributions

Chunfeng Yao: writing – original draft, data curation. Xiaoxue Wu: writing – original draft, data curation. Feiyang Dong: formal analysis, data curation. Yasen Xin: formal analysis, data curation. Zongde Wang: writing – review & editing. Shangxing Chen: validation, methodology, formal analysis. Arthur J. Ragauskas: validation, supervision, resources. Chen Huang: writing – review & editing, validation, supervision, resources. Xiuxiu Zou: writing – review & editing, validation, supervision, resources, project administration. Chunfeng Yao and Xiaoxue Wu contributed equally to this work.

## Conflicts of interest

The authors declare that they have no known competing financial interests or personal relationships that could have appeared to influence the work reported in this paper.

## Supplementary Material

RA-016-D6RA06785A-s001

## Data Availability

The original contributions presented in this study are included in the article; further inquiries can be directed to the corresponding author.
