# Supplementary material for: High strength tung oil-based adhesive with outstanding wet adhesion for cellulose fiber paper
Source: RSC Adv. 2026 Oct 7;16(46):60752–64. doi: 10.1039/d6ra06785a (PMC13644395; doi:10.1039/d6ra06785a)
Supplement: Supplementary file 1 [file RA-016-D6RA06785A-s001.pdf]

Supplementary Information (SI) for RSC Advances.
This journal is © The Royal Society of Chemistry

High strength tung oil-based adhesive with outstanding wet adhesion for cellulose fiber paper

Chunfeng Yao[a1], Xiaoxue Wu[a1], Feiyang Dong[a], Yasen Xin[a], Zongde Wang[a, b], Shangxing Chen[a], Chen Huang[c*], Arthur J. Ragauskas[d, e], Xiuxiu Zou[a*]

[a]College of Forestry, Jiangxi Agricultural University; Key Laboratory of National Forestry and Grassland Administration on Woody Fragrance and Flavor; Jiangxi Provincial Key Laboratory of Improved Variety Breeding and Efficient Utilization of Native Tree Species, Nanchang 330045, China

[b]College of Life Science, Gannan Normal University

[c]Institute of Chemical Industry of Forest Products, Chinese Academy of Forestry, Nanjing 210042, China

[d]Department of Chemical and Biomolecular Engineering, University of Tennessee Knoxville, Knoxville, TN 37996, USA

[e]Center for Renewable Carbon, School of Natural Resources, The University of Tennessee Institute of Agriculture, Knoxville, TN 37996, USA

[1] These authors should be considered joint first author.

*Corresponding author: E-mail addresses: huangchen3127@njfu.edu.cn (C. Huang), zouxiuxiuxiu@163.com (X. Zou)

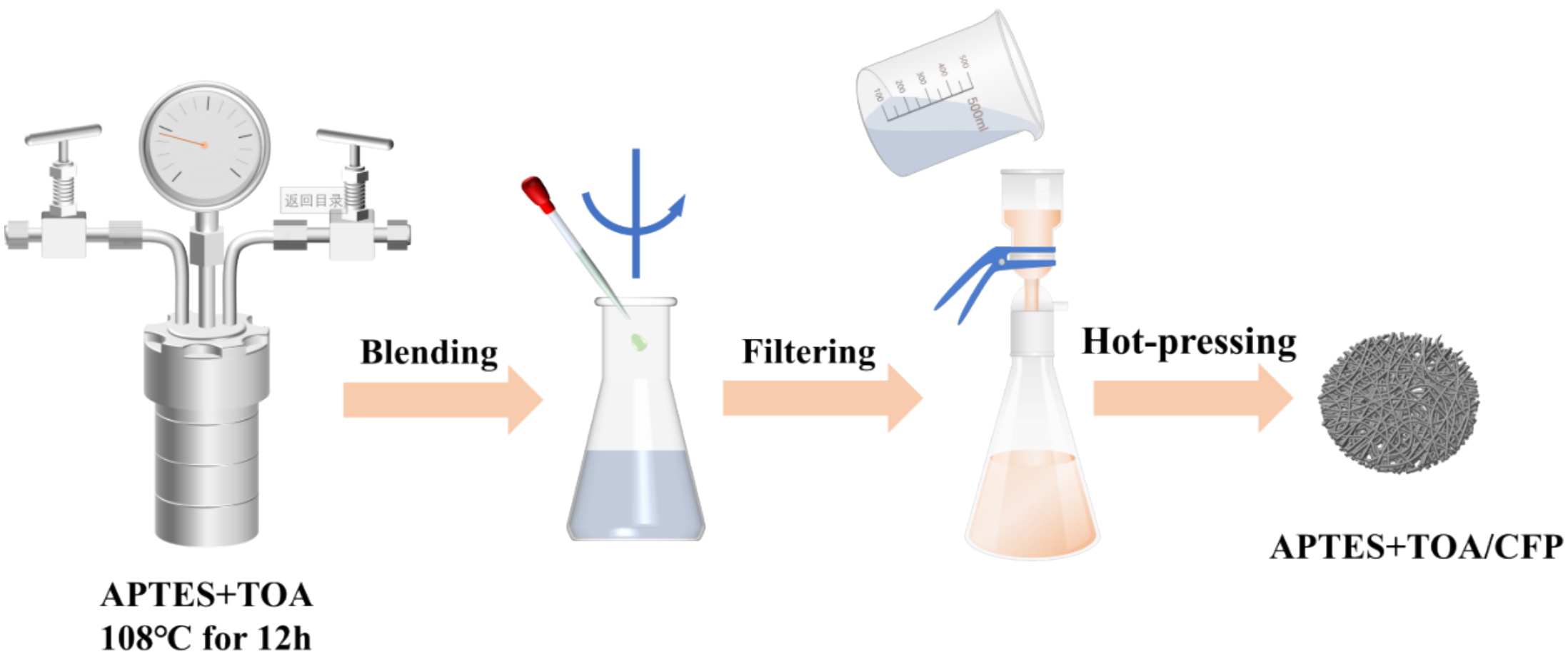

Figure S1 Schematic preparation of the synthesis process for the APTES-TOA/CFP.

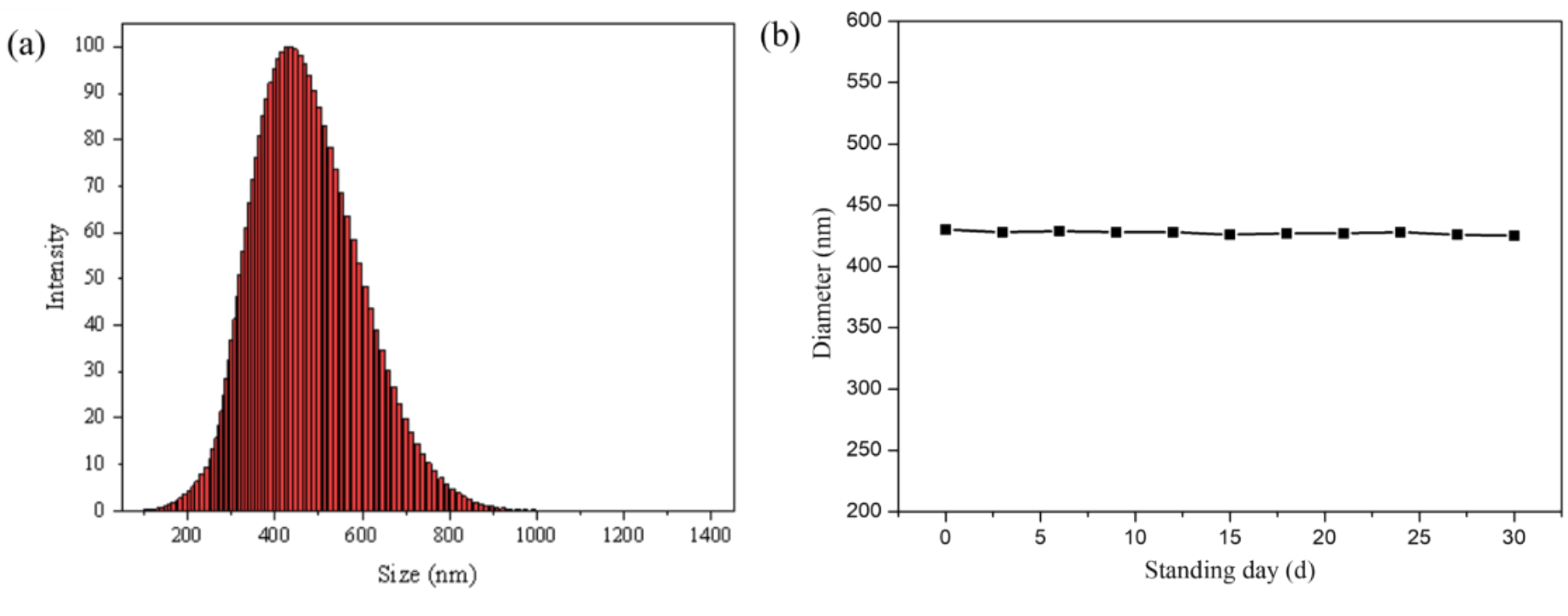

Figure S2 Diameter distribution (c) of APTES-TOA/CFP waterborne emulsion (a). The average diameter of APTES-TOA/CFP waterborne emulsion in the time range of 1 d to 30 d (b).

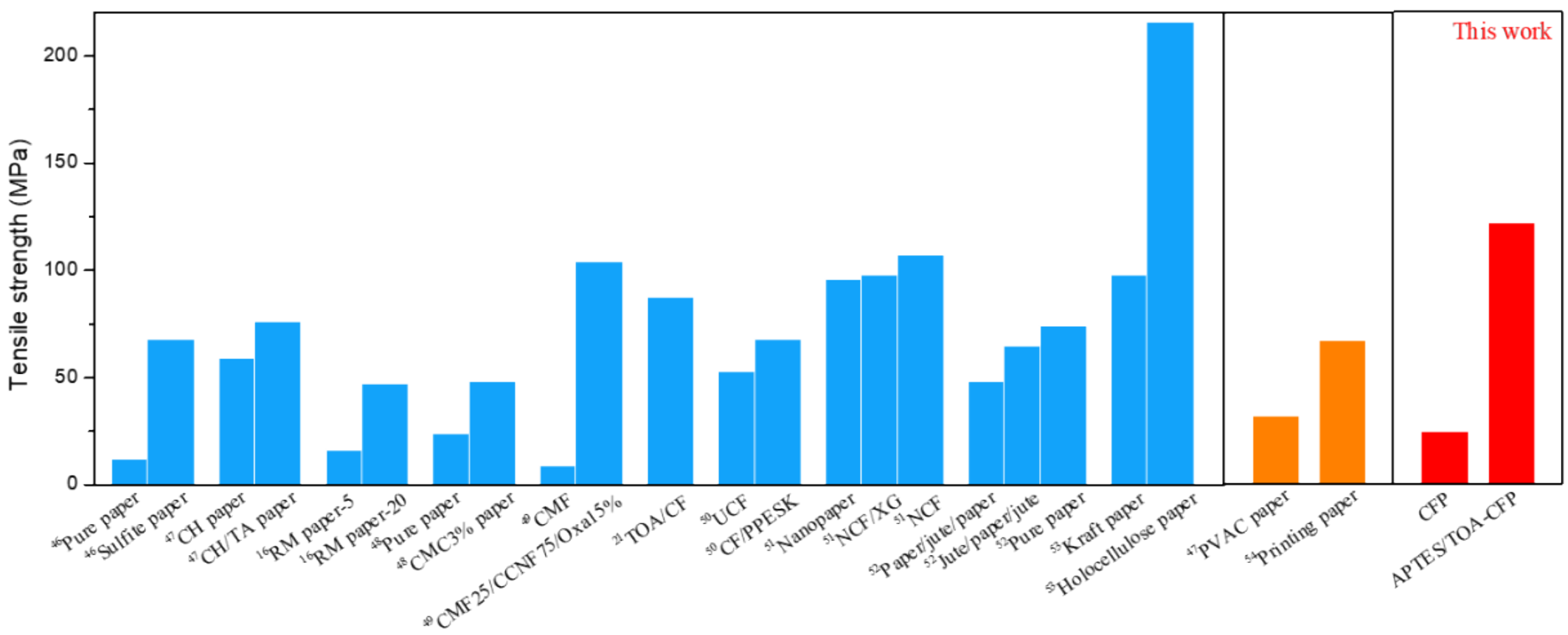

Figure S3 Comparison of the maximum tensile strength of APTES-TOA/CFP with that of other fiber cellulose paper prepared with adhesive
